# The AlzMatch Pilot Study - Feasibility of Remote Blood Collection of Plasma Biomarkers for Preclinical Alzheimer’s Disease Trials

**DOI:** 10.14283/jpad.2024.101

**Published:** 2024-06-07

**Authors:** Sarah Walter, O. Langford, G. A. Jimenez-Maggiora, S. Abdel-Latif, R. A. Rissman, J. D. Grill, J. Karlawish, A. Atri, S. Bruschi, K. Hussen, M. C. Donohue, G. A. Marshall, G. Jicha, M. Racke, R. S. Turner, C. H. van Dyck, V. Venkatesh, K. E. Yarasheski, R. Sperling, J. Cummings, P. S. Aisen, R. Raman

**Affiliations:** 1 Alzheimer’s Therapeutic Research Institute, University of Southern California, Los Angeles, USA; 2 University of California Irvine, Irvine, USA; 3 University of Pennsylvania, Philadelphia, USA; 4 Banner Sun Health Research Institute, Sun City, USA; 5 Brigham And Women’s Hospital, Massachusetts General Hospital, Harvard Medical School, Boston, USA; 6 University of Kentucky, Lexington, USA; 7 Quest Diagnostics, Secaucus, USA; 8 Georgetown University, Washington, DC, USA; 9 Yale University, New Haven, USA; 10 C2N Diagnostics, St. Louis, USA; 11 Chambers-Grundy Center for Transformative Neuroscience, Department of Brain Health, School of Integrated Health Sciences, University of Nevada, Las Vegas, USA

**Keywords:** Alzheimer’s disease, plasma biomarkers, preclinical Alzheimer’s disease, decentralized, site-agnostic methods, biomarker eligibility, community laboratories, recruitment, screening, centralized screening, remote participant engagement

## Abstract

**Background:**

Advances in plasma biomarkers to detect Alzheimer’s disease (AD) biology allows researchers to improve the efficiency of participant recruitment into preclinical trials. Recently, protein levels of plasma amyloid-beta and tau proteins have been shown to be predictive of elevated amyloid in brain. Online registries, such as the Alzheimer’s Prevention Trials (APT) Webstudy, include and follow participants using remote assessments to facilitate efficient screening and enrollment of large numbers of individuals who may be at higher risk for AD.

**Objectives:**

The AlzMatch Pilot Study investigated the feasibility of recruiting individuals from an online registry for blood sample collection at community-based phlebotomy centers and plasma biomarker quantification to assess an individual’s eligibility for AD preclinical trials.

**Design:**

Pilot feasibility study with co-primary outcomes.

**Setting:**

This pilot feasibility study included participants from the APT Webstudy, the remote assessment arm of the Trial-ready cohort for Preclinical and Prodromal AD (TRC-PAD) Platform. Novel design included collection of electronic consent, use of community laboratories for plasma collection, mass spectrometry-based biomarker assay, and telephone communication of plasma biomarker screening eligibility.

**Participants:**

Participants invited to the AlzMatch pilot feasibility study were active in the APT Webstudy, 50 years of age or older, resided within 50 miles of both a Quest Diagnostics Patient Services Center (a national diagnostic laboratory with convenient locations for sample collection and processing) and one of six TRC-PAD vanguard clinical trial sites, had no self-reported dementia diagnosis, were able to communicate in English and engaged with the APT Webstudy within the prior 6 months.

**Measurements:**

Primary feasibility outcomes were completion of electronic consent (e-consent) for invited participants and collection of usable blood samples. Additional feasibility outcomes included invitation response rate, plasma biomarker eligibility status (based on amyloid beta-42/40 [Aβ42/40] concentration ratio), ApoE proteotype, and trial inclusion criterion), and completion of telephone contact to learn eligibility to screen for a study.

**Results:**

300 APT Webstudy participants were invited to consent to the AlzMatch study. The AlzMatch e-consent rate was 39% (n=117) (95% CI of 33.5%–44.5%) overall, which was higher than the expected rate of 25%. Similar consent rates were observed across participants based on self-defined sex (41% Female (n=75), 37% Male (n=42)) and race and ethnicity (37% from underrepresented groups (URG) (n=36), 40% not from URG (n=79)). Among those that consented (n=117), plasma was successfully collected from 74% (n=87) (95% CI of 66%–82%), with similar rates across sex (76% Female (n=57), 71% Male (n=30)) and race and ethnicity (75% URG (n=27) and 75% not from URG (n=59)). 60% (n=51) of participants with plasma biomarker results were eligible to screen for future preclinical AD trials.

**Conclusion:**

Electronic consent of participants through an online registry, blood sample collection at community-based centers, plasma biomarker quantification and reporting, and biomarker assessments for study eligibility were all feasible with similar engagement rates across demographic groups. Although this pilot was a small and selective sample, participants engaged and consented at higher than expected rates. We conclude that collecting blood at community laboratories for biomarker analyses may improve accessibility beyond research, and may facilitate broader access for clinical use of AD plasma biomarkers. Based on our results, an expanded version of the AlzMatch study is underway, which involves expanding invitations to additional APT Webstudy participants and clinical trial sites.

## Introduction

**R**esearch and care for the millions of people affected by Alzheimer’s disease (AD) has entered a new phase of disease-modifying therapies (1–3). AD pathology begins to accumulate in the brain 15–20 years prior to the occurrence of symptoms (4, 5). Researchers have turned to an early stage of disease where changes in brain amyloid are present and individuals have few or no associated symptoms, known as asymptomatic or preclinical Alzheimer’s (AD) (6), as a treatment window with the most promise to delay neurodegeneration (7, 8). Identifying sufficient numbers of people with no cognitive impairment who also have elevated amyloid in brain has been a challenge, with initial trials requiring many years to complete accrual (9).

Clinical trials, and in particular AD trials, have increased in complexity of design and procedures over recent years, with negative effects on staff as well as increasing burden for participants (10, 11). From the perspective of a research participant, a number of barriers compound to limit their ability to engage in studies of preclinical AD (12). Potential participants engage in weeks or even months of screening, must be willing to learn their brain amyloid status, identify a study partner, and then undergo cognitive tests, MRI scans and either amyloid PET scans or lumbar punctures. In the case of preclinical AD studies, nearly 90% of participants ultimately learn they are not eligible despite their efforts and interest in engaging (13, 14).

Recent advances in research have provided tools that may mitigate these challenges. Decentralized research, including digital recruitment and remote/telehealth assessments, are increasingly being utilized in health intervention research, to mediate the geographical challenges and increase accessibility (15). Online registries have demonstrated broad appeal with many thousands of individuals in the US agreeing to be contacted and referred to sites conducting brain health research (16, 17). Major recent advances in the specificity, sensitivity and accuracy of blood-based biomarker tests that identify the presence or absence of brain amyloid have enabled rapid characterization and stratification of individuals who may be study candidates. Plasma amyloid beta 42/40 ratio (Aβ 42/40), p(hospho)-tau217 concentrations, p-tau217/non-p-tau217 ratio, and predictive algorithms based on these biomarkers (Amyloid Probability Score (APS1-2)) have a high concordance with amyloid PET scan results (18–21), and are now being used in pre-screening for at least one preclinical AD study (22). One prior study has evaluated feasibility of inviting individuals from the Brain Health Registry (BHR) to consent to blood collection at a community laboratory, resulting in 12% electronic consent (e-consent) and 73% collection rates. Participants for this feasibility study were invited from a single geographic region in Northern California, utilized preassembled kits, and participants were not provided any results or referred to further research (23). Leveraging both remote clinical data collection and blood-based biomarkers to identify and characterize potential participants could serve as a key strategy to accelerate clinical trial recruitment (24).

This pilot feasibility study describes the first stage of the AlzMatch program; evaluating the feasibility of blood collection and processing to plasma at community laboratories to assess eligibility of individuals for preclinical AD trials. AlzMatch builds on the successes of the Trial-Ready Cohort Program (TRC-PAD) (25). The aim of TRC-PAD is to build an efficient and sustainable recruitment system to identify, characterize, and follow individuals who are eligible for clinical trials until they enter a trial. The AlzMatch program was embedded as a part of the Alzheimer’s Prevention Trials (APT) Webstudy, the remote arm of TRC-PAD, in which participants are recruited and assessed remotely through the completion of brief quarterly assessments, which include a self-report of change in cognition, using the Cognitive Function Index (CFI), and digital cognitive testing in a subset (26, 27). All information in the APT Webstudy is self-reported by participants. The overall objective of the AlzMatch pilot study was to determine the feasibility of community-based blood collection, as a mechanism to identify participants eligible to be screened for preclinical AD studies. Primary feasibility outcomes were (1) consent rate of participants defined as completing the e-consent (target of 25%) and (2) collection rate of blood samples defined as the collection of usable plasma samples at the community-based phlebotomy centers (target of 90%). These targets were established based on prior study experience, with consideration for design differences including the broader geographic outreach and not using kits for plasma collection. Additional feasibility outcomes included the evaluation of plasma AD biomarker eligibility status (based on trial inclusion criterion), response rate to invitations, and participation rate in telephone communication of eligibility to screen for a study.

## Methods

### Ethics

The AlzMatch pilot feasibility study was conducted under a central Institutional Review Board (IRB). All participant-facing materials, (e.g. invitation emails, websites, consent forms, email and text messages) as well as the scripts used by the support team to respond to participant questions and communicate the results of plasma biomarker analyses were reviewed and approved by the central IRB, and were developed by the study’s Recruitment, Engagement and Retention team in consultation with the National Institute on Aging funded Alzheimer’s Clinical Trials Consortium (ACTC) Internal Ethics Committee.

### Disclosure of Plasma Biomarker Results

At the time of study design and implementation, limited information related to the safety and feasibility of disclosing plasma amyloid biomarker results were available. Similarly, previous protocols to deliver biomarker results included in-person education and counseling, as well as delivery of results (28). Participants were therefore informed only that their blood test result indicated whether they were ‘eligible’ or ‘not eligible’ to continue to be screened for a study. With input from the ACTC Research Participant Advisory Board, Frequently Asked Questions were prepared, to be utilized by the study team and to ensure participants were supported in these interactions.

### Plasma Collection and Analysis

Blood collection was performed at Quest Diagnostics Patient Service Centers (PSC). Quest Diagnostics was selected as a partner for this study because of their geographic reach, with over 2,000 community-based phlebotomy centers across the US, and willingness to use a research test code and standardized instructions and supplies. Blood was collected into K2 EDTA tubes, centrifuged to separate plasma, the plasma was transferred to polypropylene cryovials, frozen (at −70° to −80°C) within one hour of collection, and shipped on dry ice within 24 hours. Batches of plasma samples were shipped to C2N Diagnostics (St. Louis, MO) for Aβ42, Aβ40 concentration analyses using their validated immunoprecipitation liquid chromatography-tandem mass spectrometry platform (20). C2N’s plasma Aβ42/40 concentration ratio has been shown to have a high concordance with amyloid PET status (19–20, 29–32) and using study-specific criteria, participants were assessed for eligibility to be screened for AD research studies. Participants were not charged for the blood collection, were not required to provide insurance information, and received a $50 US dollar electronic gift card after completing the blood draw.

### Participant Journey

**Figure 1:**
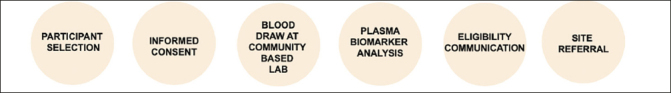
AlzMatch Participant Journey

There were six distinct phases of the participant engagement in AlzMatch, from selection of participants to be invited through the referral of plasma biomarker-eligible participants to clinical trial sites (Figure 1).

Phase 1 - Participant Selection and sampling strategy. Individuals invited to AlzMatch were selected to reflect an inclusive sample across key self-reported demographic characteristics of sex, race and ethnicity. Individuals were selected if they had consented to the APT Webstudy, were English-speaking (including bilingual English and Spanish participants), and completed at least one quarterly APT assessment in the prior 6 months. Given the imbalance observed in the APT webstudy with respect to sex (75% female) and race and/or ethnicity (90+% non-hispanic white) (27), the sampling plan aimed to balance female:male ratio to 60:40 and over sample underrepresented race and ethnic groups (URG) to understand the experience of a more inclusive sample. Individuals were excluded if they self-reported a diagnosis of dementia or were under the age of 50. To ensure participants would be able to access a study site, only individuals living within 50 miles of the six vanguard research sites received an invitation to participate. Participating sites in the AlzMatch pilot included: University of Kentucky (Lexington, KY), University of California Irvine (Irvine, CA), Yale University School of Medicine (New Haven, CT), Georgetown University (Washington DC), Brigham and Women’s Hospital (Boston, MA), and Banner Sun Health Research Institute (Phoenix, AZ).

An email was sent to selected individuals inviting them to participate in AlzMatch; reminder e-mails were sent after 7 and 14 days. Individuals who clicked the link in the email were routed to the landing page to register for AlzMatch by providing their name, email, phone and confirming they were 50 years of age or older. Registered individuals received the electronic consent by email. A support phone number was provided to individuals on the invitation emails, landing page and consent forms. Individuals had the option of receiving text message reminders in addition to e-mails.

Phase 2 - Informed Consent. The e-consent was signed by participants using Docusign. There was no requirement to speak with a member of the study team to complete e-consent, although a phone number was provided to support participants who had questions before signing. The e-consent collected key pieces of information required to generate the laboratory order at Quest: (a) Date of Birth and Name, (b) an option to provide feedback at the end of the study, and (c) an option to agree to long-term storage of their plasma sample for future research. Once e-consent was obtained, participants were sent an email with instructions for scheduling their phlebotomy appointment.

Phase 3 - Blood Draw. Participants were asked to schedule a blood draw appointment within 30 days of e-consent, and were offered the option for assistance with scheduling. Participants were emailed a laboratory order form with their AlzMatch identifier (ID). When checking in for their appointment, the laboratory order was located in the Quest system using the AlzMatch ID. This identifier was then affixed to the sample labels. After confirming the sample collection, participants were sent a thank you email with a link for a $50 electronic gift card.

Phase 4 – Potential Eligibility for AD clinical trials. Plasma samples were analyzed at C2N Diagnostics for Aβ42, Aβ40, and Aβ42/40 ratio which were reported to the AlzMatch study team for interpretation. Participant eligibility status (eligible for further screening based on increased likelihood of brain amyloid or not eligible for further screening based on a low likelihood of brain amyloid) was determined via a predictive algorithm that included the plasma Aβ42/40 results (32).

Phase 5 – Eligibility Communication. Once the eligibility status was determined, participants were contacted by phone to discuss the test results. Participants learned whether they were eligible or not eligible to continue to be screened. If eligible, participants were asked if they agreed to be referred to a research site near them. Participants not eligible were asked to continue in the APT Webstudy. After the phone call, participants were sent an email summarizing the discussion and next steps.

Phase 6 - Site Referral. Participants who agreed were referred to research sites, who then were responsible to contact participants to discuss their options for research and schedule screening visits (33). All participants referred to a research site received a follow-up phone call from the AlzMatch study team approximately 90 days after the eligibility phone call. Participants were asked to report on success of the referral, current study participation, and provided feedback, if willing to do so.

### Statistical Analysis

Demographic and additional participant characteristics were summarized using frequencies and percentages for categorical variables and using mean, standard deviation, and quartiles for continuous variables. All statistical analyses were conducted according to a pre-specified statistical analysis plan using the statistical software R (https://www.r-project.org/). Due to the exploratory nature of this study, no multiplicity adjustment was made, and results are reported using point estimates and corresponding 95% confidence intervals (CI).

Analyses of Primary Outcomes. The primary aim of the study was to estimate the event rate for the co-primary binary endpoints, namely, the consent status of an individual (i.e. consented vs. did not consent to the study), and subsequent participation in the remote blood sample draw after they consented to the study (i.e. usable blood sample vs. did not provide a sample or the sample provided was not viable). A binomial proportion confidence interval was used to calculate the point estimate of each event rate and their corresponding 95% CI. The primary analysis was performed on the overall sample and on pre-specified subgroup groups of interest, namely, participant sex, and participant race and/or ethnicity. Given the small sample sizes across the racial sub-groups, race and ethnicity were combined to two mutually exclusive groups: race and ethnic underrepresented groups (URG) (American Indian or Alaska Native race, Asian race, Black or African American race, Native Hawaiian or Other Pacific Islander race, Hispanic or Latino ethnicity) and race and ethnic non-URG (White race and Not of Hispanic ethnicity).

## Results

**Figure 2:**
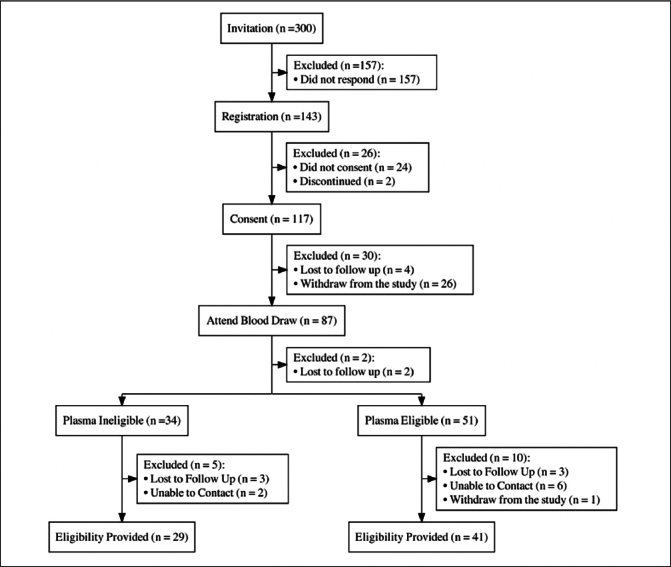
AlzMatch Pilot Consort Diagram

Figure 2 shows the flow of participants in this feasibility study. Three hundred individuals were invited from the APT Webstudy via email. Prior to consent, individuals were asked to register for AlzMatch by providing their name, email and phone number. Forty-eight percent of those invited completed this step.

### Participant Demographics

Table 1 shows the demographic characteristics of invited participants and AlzMatch e-consent outcome. 75 (64.1%) participants who completed e-consent were women. The mean age of consented participants was 64.0 years (SD 5.5). The majority of participants self-identified as White (68.7%), 9.6% identified as Asian, 0.9% identified as American Indian or Aslaka Native, 7.8% identified as Black of African American, 12.2% as Hispanic or Latino. Participants were highly educated, with only 6.8% completing 12 or less years of education, and the remainder completing some college or higher education. Only 58.1% of the participants that completed e-consent were retired, with 32.5% working full time and 9.4% working part-time. 64.1% of the participants reported a family history of Alzheimer’s disease. Participants that consented had a mean CFI score of 2.0 out of a maximum of 15, indicating some changes in cognition.

**Table 1:** AlzMatch Pilot Participant Demographics: Invited and Consent Outcome

	**Consented (N = 117)**	**Did not Consent (N = 183)**	**Total (N = 300)**
Sex			
Female	75 (64.1%)	111 (60.7%)	186 (62.0%)
Male	42 (35.9%)	72 (39.3%)	114 (38.0%)
Age			
Mean (SD)	64.0 (5.5)	64.7 (6.0)	64.4 (5.8)
Range	55.5 – 78.8	55.2 – 79.7	55.2 – 79.7
Race and Ethnicity			
Missing	2	3	5
American Indian or Alaska Native	1 (0.9%)	2 (1.1%)	3 (1.0%)
Asian	11 (9.6%)	19 (10.6%)	30 (10.2%)
Black or African American	9 (7.8%)	17 (9.4%)	26 (8.8%)
Hispanic or Latino	14 (12.2%)	22 (12.2%)	36 (12.2%)
Native Hawaiian or Other Pacific Islander	1 (0.9%)	2 (1.1%)	3 (1.0%)
White	79 (68.7%)	118 (65.6%)	197 (66.8%)
Education			
Advanced Degree	54 (46.2%)	90 (49.2%)	144 (48.0%)
College	55 (47.0%)	73 (39.9%)	128 (42.7%)
High School	8 (6.8%)	20 (10.9%)	28 (9.3%)
Employment Status			
Missing	0	2	2
Full Time	38 (32.5%)	59 (32.6%)	97 (32.6%)
Part-time	11 (9.4%)	24 (13.3%)	35 (11.7%)
Retired/Not Working	68 (58.1%)	98 (54.1%)	166 (55.7%)
Family AD/Dementia History			
Missing	0	1	1
No	42 (35.9%)	80 (44.0%)	122 (40.8%)
Yes	75 (64.1%)	102 (56.0%)	177 (59.2%)
Baseline CFI Score			
Mean (SD)	2.0 (2.0)	2.8 (2.4)	2.5 (2.3)
Range	0.0 – 10.0	0.0 – 11.0	0.0 – 11.0

### Primary Outcomes

**Figure 3:**
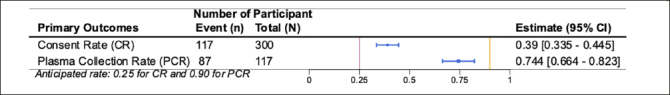
Forest plot of the primary feasibility outcomes for the AlzMatch Pilot Consent Rate (CR) and Plasma Collection Rate (PCR). The vertical bars represent the anticipated rates: 0.25 for CR and 0.90 for PCR. Each estimate is represented with a summary point and 95% confidence interval.

Results of the co-primary feasibility outcomes are presented in Figure 3. 117 out of 300 (39%; 95% CI 35% to 45%) invited individuals completed the e-consent, exceeding the anticipated e-consent rate of 25%. Eighty-seven out of the 117 (74%; 95% CI 66% to 82%) consented participants provided usable blood samples. The observed plasma collection percentage did not reach the anticipated rate of 90%. The lower than anticipated collection rate appeared to be driven by the challenges observed in the de-identification process that participants used to check in at the community laboratory.

### Other Feasibility Outcomes

We observed a high rate of sample usability (98%) with biomarker analysis completed on 85 of the 87 samples collected. A novel component of the study, namely, communicating plasma eligibility by the phone, was shown to be feasible, with 82% of participants completing this step.

### Analysis by Sub-groups

**Figure 4:**
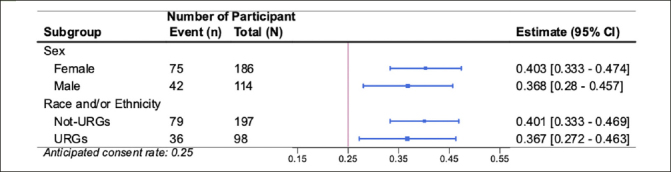
Forest plot of the subgroup analyses (participant sex and participant race and/or ethnicity underrepresented groups) for primary outcome Consent Rate (CR) to the AlzMatch study The vertical bar represents the anticipated CR of 0.25. Each estimate is represented with a summary point and 95% confidence interval.

**Figure 5:**
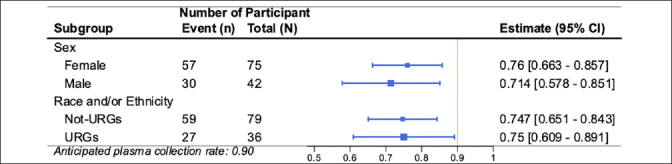
Forest plot of the subgroup analyses (participant sex and participant race and/or ethnicity underrepresented groups) for primary outcome Plasma Collection Rate (PCR) The vertical bar represents the anticipated CR of 0.25. Each estimate is represented with a summary point and 95% confidence interval.

Similar consent rates were observed across participants based on self-defined sex (41% Female, 37% Male) and race and ethnicity (37% from underrepresented groups [URG], 40% not from URG) (Figure 4). Similar plasma collection rates were also observed across participants based on self-defined sex (76% Female, 71% Male) and race and ethnicity (75% URG and 75% not from URG) (Figure 5).

### Plasma Eligibility and Referral to Research

Of the 87 blood draws, 51 (60%) participants were classified as plasma biomarker eligible, (results demonstrating a high likelihood of brain amyloid), whereas the remaining 34 (40%) participants were classified as plasma biomarker ineligible. Eligibility to be screened for research was successfully communicated to 41 (80%) of the plasma-biomarker eligible participants. The support team was unable to contact eight participants (6 plasma eligible and 2 plasma ineligible) to discuss their eligibility status after multiple attempts by phone and email. Additionally, 1 participant who was determined to be plasma eligible withdrew from the study after plasma eligibility was determined.

### Participant Withdrawal

After consent, participants were considered discontinued if they did not complete subsequent steps in the study. 26 participants completed e-consent and did not complete the blood draw, and were considered discontinued. Some participants reported inability to check in for the appointment, due to the phlebotomist being unable to locate their laboratory order. For any participant that reported issues checking in, they were provided with the $50 gift card. The full extent of these issues was not possible to measure as we did not collect data from Quest scheduling tool, which means there may have been other participants impacted by the check-in process that were not reported to the study team. 2 samples were not analyzed due to errors on sample label, 15 participants did not respond to study team outreach with their eligibility results, and were marked as ‘withdrawn’.

## Discussion

This novel pilot study demonstrated strong feasibility for identifying and consenting participants from an online AD registry for phlebotomy and plasma processing at a community laboratory. Participants engaged at a high level, the majority of samples were usable/viable for analysis, and participants who were eligible all agreed to be referred to a research site. The lower plasma collection rate observed in this AlzMatch pilot study was in large part due to issues identified in the participant check-in process at the phlebotomy locations. Given this barrier, the AlzMatch procedures were modified to implement a check-in process for participants that more closely resembled the typical workflow at the community laboratory, with central de-identification of samples.

This feasibility study, albeit preliminary with a small sample size, demonstrated no difference in rates of consent and sample collection across demographic groups, including sex, race and ethnicity. Despite having similar inclusion criteria, consent rates were higher in the AlzMatch pilot (34%) than the prior pilot study conducted by BHR (12%), including in race and ethnic underrepresented groups (URG) (23). We believe that several elements of our study design and conduct contributed to these improved results: The AlzMatch pilot study invited individuals living up to 50 miles from six clinical trials research sites across the Unites States compared to BHR inviting individuals living up to 100 miles from a single research site in Northern California. Another key difference between the studies was timing, as the BHR study took part between February and June 2020, in the height of the COVID-19 pandemic, while this study was conducted in the latter part of 2022. The AlzMatch pilot study also offered participants the opportunity to learn their eligibility for research, which was not part of the BHR pilot and may have driven higher engagement rates.

This AlzMatch pilot tested novel approaches designed to inform the future conduct of clinical trials in preclinical AD utilizing digital and remote methods, potentially providing an approach to broaden geographic access to clinical trials research. E-consent, shown to lead to greater participation in under-represented groups (34), was not a significant barrier to participation. Participants, regardless of age, sex, level of cognition, or education level, were willing to schedule and attend a blood draw at their local laboratory at similar rates. A patient-centered approach, utilizing interactive e-consent may improve these rates, or increase the number of participants that complete subsequent steps.

The feasibility of remote screening including an AD biomarker evaluation is clearly demonstrated by our data, opening up the possibility of replacing the current standard of biomarker collection at research centers to locations within one’s own community. There are several features of AlzMatch that contribute to our scalability:

1. the remote participant-driven sign up process (including invitation, e-consent and scheduling of appointment),
2. the Quest footprint with 2,000 locations in the US and use of test code instead of kits, and reduced burden on site staff by central prescreening of participants based on plasma biomarkers.

As AD clinical trials progressively replace imaging outcomes with blood-based biomarkers during the screening process (22), use of local collection methods may lead to greater research accessibility to broader groups of individuals than those who currently take part in clinical trials. Expanding research studies, including clinical trials, to include community-based blood draws and validated plasma AD biomarker analyses may reduce burden for both symptomatic and presymptomatic participants.

The learnings from this pilot have informed recruitment approaches for the widespread identification and engagement of individuals with preclinical or early-stage AD. These learnings are now being implemented in the full AlzMatch program. The present study additionally has demonstrated the feasibility of sharing eligibility results with participants by phone with follow-up email communication. The observed agreement to be referred attests to the success of the described approach. Although participants were told if they were eligible to be screened further for research, this study did not disclose to participants whether their blood test result indicated a high or low likelihood of AD pathology. Prior work has established that learning more about one’s health information is a key motivator to joining research, in particular for individuals from under-served communities (35, 36). Designing a trial that first gives participants what they want to learn and then asks about willingness to join research may yield higher engagement (37, 38). While it may be feasible to remotely disclose plasma amyloid results, the prior work emphasized the importance of participant support, choice, and ensuring results are interpreted correctly (39). A person-centered referral process from the AlzMatch study central team to research site personnel may also be needed, as not every participant chose to learn their results, and not all eligible participants were subsequently screened for research. This is an important goal of the ongoing AlzMatch program. In the future we intend to evaluate participant motivations and barriers to engage in online research, to better inform how researchers can support a successful transition from a virtual study to in-person research.

Limitations of this AlzMatch pilot study are principally due to a small sample size, thereby limiting our ability to predict how many participants would be needed to fully enroll a large multicentered clinical trial. Future AlzMatch analyses will evaluate amyloid PET status obtained subsequently in other studies for participants that were eligible based on plasma Aβ42/40 and other biomarkers, as well as discuss ideal cut-offs to use for efficient plasma biomarker prescreening. At the time of the pilot study, a validated plasma p-tau217 assay was not available. The ongoing phase of this program now incorporates p-tau217/non-phospho-tau217 measurement values in the prediction algorithm. As more plasma AD biomarkers are validated and cleared by Regulatory Authorities, predictive algorithms for brain amyloid and tau status will improve and further enhance feasibility and referral efficiencies.

There were a few factors that introduced potential participant selection bias, which is a limitation of this small pilot study. We invited participants who were enrolled in an ongoing online registry, which necessitates internet or smart phone access. We only invited participants who had completed an assessment in the past 6 months, to include the most recently engaged participants in this pilot. The third is our use of only English-language materials and support, that excludes people that do not speak English. Finally, the sample was geographically biased, as participants were only invited if they lived within 50 miles of one of six renowned academic research centers in urban areas. Ongoing community-based remote screening programs with larger sample sizes, participants who have been involved in the online registry for any length of time, expanding to monolingual Spanish participants through the use of culturally appropriate and Spanish-language outreach and participant support, and inviting people in broader geographic regions is needed to better understand the feasibility and outcomes of this approach in groups under-represented in current clinical trials (40).

## Conclusion

This AlzMatch pilot demonstrated feasibility of recruiting individuals for remote blood sample collection at community-based phlebotomy centers, plasma biomarker quantification at an accredited diagnostic laboratory, and centralized statistical prediction algorithms to assess an individual’s eligibility for AD preclinical trials. This was a small and selective sample, with strong participation and eligibility rates. Learnings from the pilot that are being implemented in the ongoing AlzMatch study include simplifying check-in procedures to better emulate typical laboratory workflow and adding the quantitation of plasma p-tau217/non-phospho-tau217 for eligibility, which was not possible to include due to the timing of the pilot study. The AlzMatch study has expanded and aims to collect 5,000 plasma samples from participants recruited from both online sources and community settings, with a focus on increasing rates of engagement from groups traditionally not represented in AD research studies. Ultimately the goal of AlzMatch is to develop inclusive methods to inform and engage participants willing to take part in studies evaluating better therapies to treat and prevent AD and related disorders. These learnings may also increase accessibility to the clinical use of AD plasma biomarkers.

## References

[1] Cummings J, Aisen P, Lemere C, Atri A, Sabbagh M, Salloway S. Aducanumab produced a clinically meaningful benefit in association with amyloid lowering. Alzheimers Res Ther. 2021;13(1):98. 33971962 10.1186/s13195-021-00838-z PMC8111757

[2] Eisai. Lecanemab Confirmatory Phase 3 Clarity AD Study Met Primary Endpoint, Showing Highly Statistically Significant Reduction Of Clinical Decline In Large Global Clinical Study Of 1,795 Participants With Early Alzheimer’s Disease. https://www.eisai.com/news/2022/news202271.html2022.

[3] Sims JR, Zimmer JA, Evans CD, et al. Donanemab in Early Symptomatic Alzheimer Disease: The TRAILBLAZER-ALZ 2 Randomized Clinical Trial. JAMA. 2023;330(6):512–527. 37459141 10.1001/jama.2023.13239 PMC10352931

[4] Li Y, Yen D, Hendrix RD, et al. Timing of Biomarker Changes in Sporadic Alzheimer’s Disease in Estimated Years from Symptom Onset. Ann Neurol. 2024. 10.1002/ana.26891 PMC11060905 38400792

[5] Jia J, Ning Y, Chen M, et al. Biomarker Changes during 20 Years Preceding Alzheimer’s Disease. N Engl J Med. 2024;390(8):712–722. 38381674 10.1056/NEJMoa2310168

[6] Sperling RA, Aisen PS, Beckett LA, et al. Toward defining the preclinical stages of Alzheimer’s disease: recommendations from the National Institute on Aging-Alzheimer’s Association workgroups on diagnostic guidelines for Alzheimer’s disease. Alzheimers Dement. 2011;7(3):280–292. 21514248 10.1016/j.jalz.2011.03.003 PMC3220946

[7] Rafii MS, Sperling RA, Donohue MC, et al. The AHEAD 3–45 Study: Design of a prevention trial for Alzheimer’s disease. Alzheimers Dement. 2022. 10.1002/alz.12748 PMC9929028 35971310

[8] A Donanemab (LY3002813) Prevention Study in Participants With Alzheimer’s Disease (TRAILBLAZER-ALZ 3). ClinicalTrials.gov2024.

[9] Sperling RA, Donohue MC, Raman R, et al. Association of Factors With Elevated Amyloid Burden in Clinically Normal Older Individuals. JAMA Neurol. 2020;77(6):735–745. 32250387 10.1001/jamaneurol.2020.0387 PMC7136861

[10] Getz KA, Campo RA. New Benchmarks Characterizing Growth in Protocol Design Complexity. Ther Innov Regul Sci. 2018;52(1):22–28. 29714620 10.1177/2168479017713039

[11] Goldman D, Malzbender K, Lavin-Mena L, Hughes L, Bose N, Patel D. Key Barriers for Clinical Trials for Alzheimer’s Disease. 2020; https://healthpolicy.usc.edu/research/key-barriers-for-clinical-trials-for-alzheimers-disease/. Accessed 1/5/2024, 2024.

[12] Grill JD, Zhou Y, Elashoff D, Karlawish J. Disclosure of amyloid status is not a barrier to recruitment in preclinical Alzheimer’s disease clinical trials. Neurobiol Aging. 2016;39:147–153. 26923411 10.1016/j.neurobiolaging.2015.11.007 PMC4773920

[13] Manly JJ, Gilmore-Bykovskyi A, Deters KD. Inclusion of Underrepresented Groups in Preclinical Alzheimer Disease Trials-Opportunities Abound. JAMA Netw Open. 2021;4(7):e2114606. 34228130 10.1001/jamanetworkopen.2021.14606

[14] Grill JD, Raman R, Ernstrom K, Aisen P, Karlawish J. Effect of study partner on the conduct of Alzheimer disease clinical trials. Neurology. 2013;80(3):282–288. 23255824 10.1212/WNL.0b013e31827debfe PMC3589183

[15] Goodson N, Wicks P, Morgan J, Hashem L, Callinan S, Reites J. Opportunities and counterintuitive challenges for decentralized clinical trials to broaden participant inclusion. NPJ Digit Med. 2022;5(1):58. 35513479 10.1038/s41746-022-00603-y PMC9072305

[16] Langbaum JB, Karlawish J, Roberts JS, et al. GeneMatch: A novel recruitment registry using at-home APOE genotyping to enhance referrals to Alzheimer’s prevention studies. Alzheimers Dement. 2019;15(4):515–524. 30772251 10.1016/j.jalz.2018.12.007 PMC6461487

[17] Langbaum JB, High N, Nichols J, Kettenhoven C, Reiman EM, Tariot PN. The Alzheimer’s Prevention Registry: A Large Internet-Based Participant Recruitment Registry to Accelerate Referrals to Alzheimer’s-Focused Studies. J Prev Alzheimers Dis. 2020;7(4):242–250. 32920626 10.14283/jpad.2020.31 PMC7534299

[18] Schindler SE, Bollinger JG, Ovod V, et al. High-precision plasma β-amyloid 42/40 predicts current and future brain amyloidosis. Neurology. 2019;93(17):e1647–e1659. 31371569 10.1212/WNL.0000000000008081 PMC6946467

[19] Rissman RA, Langford O, Raman R, et al. Plasma Aβ42/Aβ40 and phosphotau217 concentration ratios increase the accuracy of amyloid PET classification in preclinical Alzheimer’s disease. Alzheimers Dement. 2024;20(2):1214–1224. 37932961 10.1002/alz.13542 PMC10916957

[20] Hu Y, Kirmess KM, Meyer MR, et al. Assessment of a Plasma Amyloid Probability Score to Estimate Amyloid Positron Emission Tomography Findings Among Adults With Cognitive Impairment. JAMA Netw Open. 2022;5(4):e228392. 35446396 10.1001/jamanetworkopen.2022.8392 PMC9024390

[21] Meyer MR, Kirmess KM, Eastwood S, et al. Clinical validation of the PrecivityAD2 blood test: A mass spectrometry-based test with algorithm combining %p-tau217 and Aβ42/40 ratio to identify presence of brain amyloid. Alzheimers Dement. 2024; 1–14. doi:10.1002/alz.13764 10.1002/alz.13764 PMC11095426 38491912

[22] Sperling R, Johnson K, Zhou J, et al. Introduction of Plasma Biomarker Screening for the AHEAD 3–45 Study. presented at: CTAD2021.

[23] Fockler J, Ashford MT, Eichenbaum J, et al. Remote blood collection from older adults in the Brain Health Registry for plasma biomarker and genetic analysis. Alzheimers Dement. 2022;18(12):2627–2636. 35226409 10.1002/alz.12617 PMC9998146

[24] Langbaum JB, Zissimopoulos J, Au R, et al. Recommendations to address key recruitment challenges of Alzheimer’s disease clinical trials. Alzheimers Dement. 2022. 10.1002/alz.12737 PMC9911558 35946590

[25] Aisen PS, Sperling RA, Cummings J, et al. The Trial-Ready Cohort for Preclinical/Prodromal Alzheimer’s Disease (TRC-PAD) Project: An Overview. J Prev Alzheimers Dis. 2020;7(4):208–212. 32920621 10.14283/jpad.2020.45 PMC7735207

[26] Walter S, Clanton TB, Langford OG, et al. Recruitment into the Alzheimer Prevention Trials (APT) Webstudy for a Trial-Ready Cohort for Preclinical and Prodromal Alzheimer’s Disease (TRC-PAD). J Prev Alzheimers Dis. 2020;7(4):219–225. 32920623 10.14283/jpad.2020.46 PMC7842199

[27] Walter S, Langford OG, Clanton TB, et al. The Trial-Ready Cohort for Preclinical and Prodromal Alzheimer’s Disease (TRC-PAD): Experience from the First 3 Years. J Prev Alzheimers Dis. 2020;7(4):234–241. 32920625 10.14283/jpad.2020.47 PMC7767585

[28] Grill JD, Raman R, Ernstrom K, Sultzer DL, Burns JM, Donohue MC, Johnson KA, Aisen PS, Sperling RA, Karlawish J; A4 Study Team. Short-term Psychological Outcomes of Disclosing Amyloid Imaging Results to Research Participants Who Do Not Have Cognitive Impairment. JAMA Neurol. 2020 Dec 1;77(12):1504–1513. 32777010 10.1001/jamaneurol.2020.2734 PMC7418046

[29] Langford O, Raman R, Sperling RA, et al. Predicting Amyloid Burden to Accelerate Recruitment of Secondary Prevention Clinical Trials. J Prev Alzheimers Dis. 2020;7(4):213–218. 32920622 10.14283/jpad.2020.44 PMC7745538

[30] Fogelman I, West T, Braunstein JB, et al. Independent study demonstrates amyloid probability score accurately indicates amyloid pathology. Ann Clin Transl Neurol. 2023;10(5):765–778. 36975407 10.1002/acn3.51763 PMC10187729

[31] West T, Kirmess KM, Meyer MR, et al. A blood-based diagnostic test incorporating plasma Aβ42/40 ratio, ApoE proteotype, and age accurately identifies brain amyloid status: findings from a multi cohort validity analysis. Mol Neurodegener. 2021;16(1):30. 33933117 10.1186/s13024-021-00451-6 PMC8088704

[32] Rissman RA, Langford O, Raman R, et al. Plasma Aβ42/Aβ40 and phosphotau217 concentration ratios increase the accuracy of amyloid PET classification in preclinical Alzheimer’s disease. Alzheimers Dement. 2023. 10.1002/alz.13542 PMC10916957 37932961

[33] Jimenez-Maggiora GA, Bruschi S, Raman R, et al. TRC-PAD: Accelerating Recruitment of AD Clinical Trials through Innovative Information Technology. J Prev Alzheimers Dis. 2020;7(4):226–233. 32920624 10.14283/jpad.2020.48 PMC7769128

[34] Fanaroff AC, Li S, Webb LE, et al. An Observational Study of the Association of Video- Versus Text-Based Informed Consent With Multicenter Trial Enrollment: Lessons From the PALM Study (Patient and Provider Assessment of Lipid Management). Circ Cardiovasc Qual Outcomes. 2018;11(4):e004675. 29625993 10.1161/CIRCOUTCOMES.118.004675 PMC5891825

[35] Erickson CM, Chin NA, Ketchum FB, et al. Predictors of Willingness to Enroll in Hypothetical Alzheimer Disease Biomarker Studies that Disclose Personal Results. Alzheimer Dis Assoc Disord. 2022;36(2):125–132. 35125399 10.1097/WAD.0000000000000490 PMC9132241

[36] Ketchum FB, Erickson CM, Chin NA, et al. What Influences the Willingness of Blacks and African Americans to Enroll in Preclinical Alzheimer’s Disease Biomarker Research? A Qualitative Vignette Analysis. Journal of Alzheimer’s Disease. 2022;87:1167–1179. 35466937 10.3233/JAD-215521 PMC9198766

[37] Walter S, Boxer A, Grill, Joshua D., Karlawish, Jason, Shaffer, Elizabeth, Ziolkowski, Jaimie, Aisen, Paul S. Preference for Disclosure of Biomarker and Genetic Results in Alzheimer’s Research in Alzheimer’s Research: Feedback from a Participant Advisory Board. The Journal of Prevention of Alzheimer’s Disease. 2021;8(1):S73–S170.

[38] Walter S, Taylor A, Tyrone J, et al. Disclosing Individual Results in Dementia Research: A Proposed Study Participant’s Bill of Rights. J Alzheimers Dis. 2022;90(3):945–952. 36278354 10.3233/JAD-220810 PMC10120612

[39] Erickson CM, Chin NA, Rosario HL, Peterson A, Johnson SC, Clark LR. Feasibility of virtual Alzheimer’s biomarker disclosure: Findings from an observational cohort. Alzheimers Dement (N Y). 2023;9(3):e12413. 37521522 10.1002/trc2.12413 PMC10382796

[40] Mindt MR, Okonkwo O, Weiner MW, et al. Improving generalizability and study design of Alzheimer’s disease cohort studies in the United States by including under-represented populations. Alzheimers Dement. 2022. 10.1002/alz.12823 PMC10101866 36372959

